# Akt Kinase Intervenes in Flavivirus Replication by Interacting with Viral Protein NS5

**DOI:** 10.3390/v13050896

**Published:** 2021-05-12

**Authors:** Laura Albentosa-González, Nereida Jimenez de Oya, Armando Arias, Pilar Clemente-Casares, Miguel Ángel Martin-Acebes, Juan Carlos Saiz, Rosario Sabariegos, Antonio Mas

**Affiliations:** 1 Unidad de Medicina Molecular, Centro Regional de Investigaciones Biomédicas, Universidad de Castilla-La Mancha, 02008 Albacete, Spain; laura.albentosa@alu.uclm.es (L.A.-G.); Armando.Arias@uclm.es (A.A.); Pilar.CCasares@uclm.es (P.C.-C.); 2 ZOOVIR, Department of Biotechnology, Instituto Nacional de Investigación y Tecnología Agraria y Alimentaria, 28040 Madrid, Spain; adierenji@gmail.com (N.J.d.O.); martin.mangel@inia.es (M.Á.M.-A.); jcsaiz@inia.es (J.C.S.); 3 Unidad de Biomedicina UCLM-CSIC, 02008 Albacete, Spain; 4 Escuela Técnica Superior de Ingenieros Agrónomos, Universidad de Castilla-La Mancha, 02071 Albacete, Spain; 5 Facultad de Farmacia, Universidad de Castilla-La Mancha, 02008 Albacete, Spain; 6 Facultad de Medicina, Universidad de Castilla-La Mancha, 02008 Albacete, Spain

**Keywords:** flavivirus, replicase, NS5, RNA-dependent RNA-polymerase, PI3K/Akt/mTOR pathway, inhibitors, host factors

## Abstract

Arthropod-borne flaviviruses, such as Zika virus (ZIKV), Usutu virus (USUV), and West Nile virus (WNV), are a growing cause of human illness and death around the world. Presently, no licensed antivirals to control them are available and, therefore, search for broad-spectrum antivirals, including host-directed compounds, is essential. The PI3K/Akt pathway controls essential cellular functions involved in cell metabolism and proliferation. Moreover, Akt has been found to participate in modulating replication in different viruses including the flaviviruses. In this work we studied the interaction of flavivirus NS5 polymerases with the cellular kinase Akt. In vitro NS5 phosphorylation experiments with Akt showed that flavivirus NS5 polymerases are phosphorylated and co-immunoprecipitate by Akt. Polymerase activity assays of Ala- and Glu-generated mutants for the Akt-phosphorylated residues also indicate that Glu mutants of ZIKV and USUV NS5s present a reduced primer-extension activity that was not observed in WNV mutants. Furthermore, treatment with Akt inhibitors (MK-2206, honokiol and ipatasertib) reduced USUV and ZIKV titers in cell culture but, except for honokiol, not WNV. All these findings suggest an important role for Akt in flavivirus replication although with specific differences among viruses and encourage further investigations to examine the PI3K/Akt/mTOR pathway as an antiviral potential target.

## 1. Introduction

Flaviviruses are mosquito-borne arboviruses (**ar**thropod-**bo**rne **v**iruses) of the *Flaviviridae* family that included four genera (*Flavivirus*, *Hepacivirus*, *Pegivirus*, and *Pestivirus*). *Flavivirus* is the largest genus with a significant number of species causing pathology in humans and other animals that are relevant to public health, such as dengue virus (DENV), yellow fever virus (YFV), Japanese encephalitis virus (JEV), West Nile virus (WNV), Zika virus (ZIKV) and Usutu virus (USUV) [1]. Some of these pathogens can be contained through vaccination with high levels of protection achieved (JEV, YFV) [1]. Although some drugs have been approved for the treatment of infection [2,3], most flavivirus diseases, including those responsible for large epidemics (DENV, ZIKV), are not treatable, and only palliative approaches aimed at reducing the symptoms are available [4].

Positive ssRNA viruses show high mutation and replication rates which enable their rapid adaptation to the environment, and developing resistance to the presence of an antiviral drug or to the host’s immune response [5]. An alternative antiviral approach to avoid the rapid emergence of resistance mutants is to target the cellular pathways necessary for the virus to replicate [6,7]. In this scenario, the emergence of resistant mutant viruses is even less likely as the drug targets a protein coded by the host cell genome.

Flavivirus genome is composed of a single-stranded RNA genome of positive polarity ((+)ssRNA) that is translated as a single polyprotein that, after being processed by viral and host proteases, gives rise to three structural (capsid, pre-membrane, and envelope) and 7 non-structural proteins (NS1, NS2A, NS2B, NS3, NS4A, NS4B, and NS5) [8]. NS5 is the most conserved flavivirus proteins with at least 68% amino acid identity for WNV, ZIKV and USUV NS5 proteins [9]. NS5 is a multifunctional protein that contains an N-terminal methyltransferase domain, and the RNA-dependent RNA-polymerase (RdRp) activity in the C-terminus region.

Previous work by our group has shown that the PI3K/Akt/mTOR pathway is involved in the replication of hepatitis C virus (HCV), a virus of the genus *Hepacivirus*. We described that the HCV polymerase, namely NS5B, interacts and colocalizes with the Akt cell kinase. In addition, we showed that an Akt-specific inhibitor (MK-2206) inhibits virus replication in cell culture [10]. Owing to these precedents, we were interested in characterizing whether members of *genus Flavivirus* (USUV, WNV and ZIKV), which are genetically related to the genus *Hepacivirus*, are also regulated by Akt.

We found that USUV, WNV and ZIKV NS5 proteins are phosphorylated by Akt in vitro. This post-translational modification affected the RNA-polymerase activity of NS5 proteins. We further confirmed the interaction of these NS5 proteins with Akt in the host cell, and that specific Akt drugs inhibit the replication of USUV and ZIKV in tissue culture.

## 2. Materials and Methods

### 2.1. DNA Amplification by PCR and Cloning

**Figure 1:**
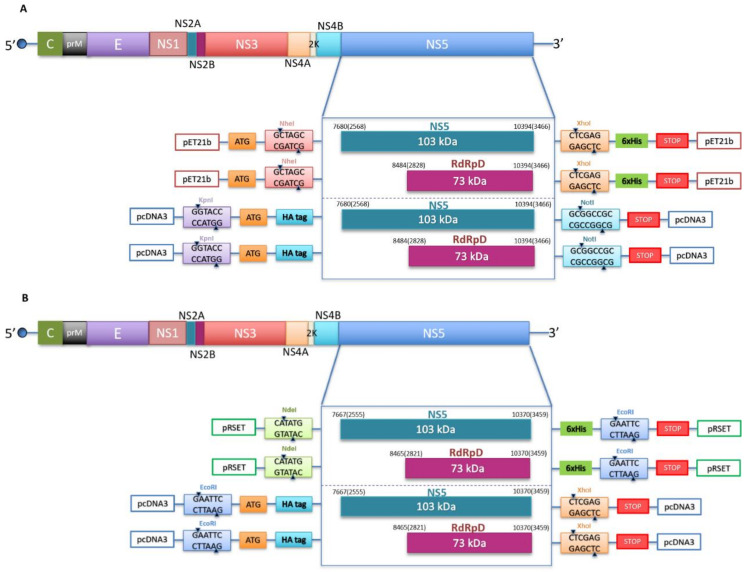
Schematic representation of the constructs used in this study. (**A**) WNV genome organization showing the polyprotein coding region (above) and the cloning strategy for the constructs used in this study (below). Two different inserts (NS5 and RdRp domain) were introduced into two different vectors, pET21b with the cloning sites for NheI (GCTAGC) and XhoI (CTCGAG) and a 6xHis tag, and pcDNA3 with the cloning sites for KpnI (GGTACC) and NotI (GCGGCCGC) and an HA tag in the N-terminus. Numbers indicate the positions of nucleotide and amino acid residues (in brackets) of the N-terminal and C-terminal ends of NS5 and RdRp domain. The numbering refers to WNV strain in wild birds in Serbia (GenBank accession number KC407673.1). (**B**) ZIKV genome organization showing the polyprotein coding region (above) and the cloning strategy for the constructs used in this study (below). Two different inserts (NS5 and RdRp domain) are introduced into two different vectors, pRSET with the cloning sites for NdeI (CATATG) and EcoRI (GAATTC), and a 6xHis tag, and pcDNA3 with the cloning sites for EcoRI (GAATTC) and XhoI (CTCGAG), and an HA tag in the N-terminal end. Numbers are referring to the positions of nucleotide and amino acid residues (in brackets) of the N-terminal and C-terminal ends of NS5 and RdRp domain. The numbering refers to ZIKV strain (GenBank accession number KX377337.1).

WNV (SRB Novi-Sad/12, lineage 2, KC407673.1) and ZIKV (PRVABC59, Puerto Rico, 2015; ATCC reference number VR-1843) sequences encoding for the entire NS5 protein and its RdRp domain (RdRpD) were amplified by PCR, using specifically designed primers (Table 1), PfuTurbo DNA polymerase (Agilent Technologies, Santa Clara, CA, USA), and a plasmid containing a fragment of the WNV and ZIKV sequence from which we extracted the coding sequences as a template. The NS5 and RdRpD fragments were cloned into eukaryotic pcDNA3 (Thermo Fisher Scientific, Waltham, MA, USA) and bacterial pET21b/pRSET (Novagen, Madison, USA) expression vectors using the restriction enzyme sites included in the primers. A human influenza hemagglutinin (HA) epitope was incorporated into the forward primer of each pcDNA3 construct. The constructs contain a 6x-His tag at their C-termini included in the vector (Table 1 and Figure 1) for NS5 expression. Residues Ser664, Ser669 and Ser670 of ZIKV, USUV and WNV NS5, respectively, were substituted by Glu (Mut E) or Ala (Mut A) by site-directed mutagenesis (Quikchange Lightning Site-Directed Mutagenesis kit-Agilent Technologies, Santa Clara, CA, USA, using the NS5 pET21b/pRSET construct as a template. Primers for mutagenesis are described in Table 1. The expected sequence was confirmed by DNA sequencing for all the constructs. Plasmids encoding the entire NS5 protein and the RdRpD of USUV have been previously described [11].

### 2.2. NS5 and RdRpD Protein Purification

pET21b and pRSET plasmid constructs were used to transform the BL21(DE3) pLysS Rosetta *Escherichia coli* strain. Protein purification procedures have been previously described [10,11,12,13]. 

### 2.3. In Vitro Kinase Activity Assay

NS5 proteins (1 μg) were incubated in hot kinase buffer (20 mM Hepes pH 7.4, 10 mM MgCl_2_, 10 mM MnCl_2_, 1 μCi of [γ-^32^P] ATP, 1 mM DTT) in the presence of 0.5 μg of recombinant Akt/PKB (Biaffin GmbH&Co, Kassel, Germany) in a final volume of 30 µL. The products were separated on an SDS-PAGE gel. After electrophoresis, the gel was dried, exposed to a phosphorimager screen and scanned in a Typhoon9600 device (Molecular Dynamics).

### 2.4. Proteomic Analyses

NS5 proteins (3 μg) were incubated in kinase buffer (20 mM Hepes pH 7.4, 10 mM MgCl_2_, 10 mM MnCl_2_, 1 mM DTT) in the presence of 0.5 μg of recombinant Akt/PKB (Biaffin GmbH&Co). The protein extracts were suspended in a volume up to 50 µL of sample buffer, and then applied onto 1.2-cm wide wells of a conventional SDS-PAGE gel (1.5 mm-thick, 4% stacking, and 10% resolving).

The gel could run long enough for all protein bands to resolve well on the gel. Coomassie staining was performed. The gel was fixed for 30 minutes in a solution of 50% methanol 10% acetic acid.

The separated protein bands were, excised, cut into cubes (2 × 2 mm), and placed in 0.5 mL microcentrifuge tubes [14]. The gel pieces were destained in acetonitrile: water (ACN:H2O, 1:1), reduced and alkylated (disulfide bonds from cysteinyl residues were reduced with 10 mM DTT for 1 h at 56 °C, and then thiol groups were alkylated with 10 mM iodoacetamide for 30 min at room temperature in darkness), and digested in situ with sequencing grade trypsin (Promega, Madison, WI, USA) and chymotrypsin (Roche, Mannheim, Germany) as described by Shevchenko et al., [15] with minor modifications. The gel pieces were shrunk by removing all liquid using sufficient ACN. Acetonitrile was pipetted out and the gel pieces were dried in a speedvac. The dried gel pieces were re-swollen in 100 mM TrisHCl pH 8, 10 mM CaCl2 with 60 ng/µL trypsin or chymotrypsin at 5:1 protein:enzyme (*w*/*w*) ratio. The tubes were kept on ice for 2 h and incubated at 37 °C (trypsin) or 25 °C (chymotrypsin) for 12 h. Digestion was stopped by the addition of 1% TFA. Whole supernatants were dried down and then desalted onto ZipTip C18 Pipette tips (Millipore) until the mass spectrometric analysis. 

The desalted protein digest was dried, resuspended in 10 µL of 0.1% formic acid and analyzed by RP-LC-MS/MS in an Easy-nLC II system coupled to an ion trap LTQ Orbitrap-Velos-Pro hybrid mass spectrometer (Thermo Scientific, Waltham, MA, USA). The peptides were concentrated (online) by reverse phase chromatography using a 0.1mm × 20 mm C18 RP precolumn (Thermo Scientific), and then separated using a 0.075mm × 250 mm C18 RP column (Thermo Scientific) operating at 0.3 μL/min. Peptides were eluted using a 90-min dual gradient. The gradient profile was set as follows: 5−25% solvent B for 68 min, 25−40% solvent B for 22 min, 40−100% solvent B for 2min and 100% solvent B for 18 min (Solvent A: 0.1% formic acid in water, solvent B: 0.1% formic acid, 80% acetonitrile in water). ESI ionization was done using a Nano-bore emitters Stainless Steel ID 30 μm (Proxeon) interface at 2.1 kV spray voltage with S-Lens of 60%. The Orbitrap resolution was set at 30,000 [16]. Peptides were detected in survey scans from 400 to 1600 amu (1 μscan), followed by 15 data-dependent MS/MS scans (Top 15), using an isolation width of 2 u (in mass to-charge ratio units), normalized collision energy of 35%, dynamic exclusion applied during 60 s periods and MSA (Multistep activation). Charge-state screening was enabled to reject unassigned and singly charged protonated ions. If the sequence of any peptide of interest is known, the mass spectrometer was further operated in the selected MS/MS ion monitoring mode (SMIM mode) [17]. In this mode, the LTQ-Orbitrap-Velos-Pro detector was programmed to perform, along the same entire gradient, a continuous sequential operation in the MS/MS mode on the doubly or triply charged ions corresponding to the peptide/s selected previously from the theoretical prediction. 

Peptide identification from raw data was carried out using the SEQUEST algorithm (Proteome Discoverer 2.2, Thermo Scientific) or PEAKS Studio X+ search engine (Bioinformatics Solutions Inc., Waterloo, Ontario, Canada). Database search was performed against a Local Database with the protein sequences of interest. The following constraints were used for the searches: tryptic cleavage after Arg and Lys or chymotryptic cleavage after Tyr, Trip, Phe, up to two missed cleavage sites, and tolerances of 20 ppm for precursor ions and 0.6 Da for MS/MS fragment ions and the searches were performed allowing optional Ser, Thr and Tyr phosphorylation, Met oxidation and Cys carbamidomethylation. False discovery rates (FDR) for peptide spectrum matches (PSM) were limited to 0.01. Only those proteins with at least two distinct peptides and at least one unique peptide being discovered from LC/MS/MS analyses were considered reliably identified. Protein Identification and characterization by LC-MS/MS was carried out in the ‘CBMSO PROTEIN CHEMISTRY FACILITY’, which belongs to ProteoRed, PRB3-ISCIII, supported by grant PT17/0019, of the PE I+D+i 2013–2016, funded by ISCIII and ERDF. 

### 2.5. In Vitro RdRp Activity Assays

Polymerase activity tests to detect de novo activity was performed as previously described using an RNA substrate mimicking the 3′ end of USUV negative strand USUV20 (Table 1) [12]. 

Polymerase activity tests to detect primer-extension activities were performed in a primer-extension reaction using a fluorescently labeled RNA primer-RNA template complex (P/T) as previously described [18] with some modifications. The P/T complex was obtained by mixing 1 µM fluorescently labeled oligonucleotide primer (Cy5.5-5′-AGAACCUGUUGAACAAAAGC-3′) and 4 µM unlabeled RNA template (5′-CUUAUUCGAGCUUUUGUUCAACAGGUUCU-3′) in 50 mM NaCl. The mixture was incubated at 95 °C for 10 min and slowly cooled (1 °C/min) to 20 °C. The primer-extension reaction contained 5 mM MOPS pH 7.25 (5 mM Tris-HCl pH 7.5 for ZIKV enzymes), 10 mM DTT, 5 mM MnCl2, 0.5% Triton X-100, 10% glycerol, 10 nM P/T and NS5 protein at the concentration indicated in each experiment. The reaction was initiated by the addition of rNTPs at a final concentration of 100 µM. The reaction proceeded at 35 °C, and then it was stopped at different time points by the addition of a 2× EDTA/formamide loading buffer. Double stranded RNA reaction products were denatured by incubating the samples at 95 °C for 15 min and resolved by electrophoresis on a 23% denaturing polyacrylamide gel (urea-PAGE) in Tris-borate-EDTA buffer (TBE). After electrophoresis, the gels were scanned using a Typhoon imaging system. The images were analyzed, and the elongation products quantified using the Quantity One 1-D analysis software Version 4.6.5.

To analyze polymerase activity over time, we used 100 nM of each enzyme (WT, Mut E and Mut A). WNV WT was also assayed at 500 nM. Reactions were set up and performed as described above. Aliquots of the reaction were collected at 0, 2, 4, 6, 8, 10, 20 and 30 min for most of the enzymes. USUV mut E was analyzed at 0, 4, 8, 10, 20, 30, 60 and 120 min and ZIKV Mut E at 0, 2, 4, 6, 8, 10, 20, 30 and 60 min.

To test if the presence of AKT/PKB may be affecting the RdRp activity of NS5, 400 nM WNV NS5 WT was incubated in 5mM MOPS pH 7, 25, 10 mM DTT, 5 mM MnCl_2_, 0.5% Triton X-100, 10% glycerol alone or supplemented with 0.5 µg Akt (Biaffin GmbH & Co) or 0.5 µg Akt plus 500 µM ATP for 30 min at room temperature. After incubation, the reaction was started by adding 100 µM NTP and 10 nM T/P and stopped after 6 min. Reactions were resolved in 23% denaturing polyacrylamide gel electrophoresis (urea-PAGE) in TBE buffer.

### 2.6. Cell Culture, Western Blot, Co-Immunoprecipitation and Treatment

For Western blot (WB) and co-immunoprecipitation (Co-IP) assays we used human cell lines Huh7.5 and Hek-293T. WB and Co-IP procedures have been previously described [11]. As a control prior to the co-immunoprecipitation experiment we used anti-GFP antibodies to check that we did not rescue Akt, NS5 or the RdRp domain from the immunoprecipitate.

Cytotoxicity was evaluated as previously described [11]. Briefly, we used 96-well plates seeded with Vero cells as above to determine the relative number of viable cells after treatment with MK-2206, ipatasertib and honokiol. Subsequently, we treated the cell monolayers with increasing concentrations of the drug, between 5 and 40 µM.

At 48 h after drug administration, 20 µL of CellTiter-Blue Cell Reagent (CellTiter-Blue cell viability assay kit) were added to each well, according to the manufacturer’s instructions (Promega, Madison, WI, USA). The cells were then incubated at 37 °C, and the fluorescence emitted was recorded between 30 and 120 min later. The number of live metabolically active cells in the culture is directly proportional to the fluorescence detected.

To analyze the antiviral effect of PI3K/AKT/mTOR inhibitors, cells were supplemented with 1 mL of media containing 1% FBS and 5–10 µM of ipatasertib, MK-2206, or honokiol in the conditions described in the text. All compounds were purchased from Selleckchem. Lineage 2 WNV strain WNV SRB Novi Sad/12 (GenBank accession number: KC407673) isolated from a dead goshawk in Serbia in 2012 [19], and ZIKV strain PRVABC59, isolated in Puerto Rico (ATCC reference number VR-1843) were used as previously described [20,21]. Vero cells were treated with in the presence (pretreated) or absence (not pretreated) of Akt drugs at the indicated concentrations in 24-well plates. After 5 h of incubation, ZIKV or WNV was inoculated at a multiplicity of infection (MOI) of 2 and virus adsorption was allowed for 1 h. Then, the cells were extensively washed to remove any unbound virus particles, and 1 mL of media containing 1% FBS and the corresponding Akt inhibitor was added to each well. Culture supernatant samples were collected at different post-inoculation times and titrated as previously described for ZIKV [20] and WNV [21].

### 2.7. Statistical Analyses

Statistical significance was examined using GraphPad Prism 8 as described in the figure legends, and following the recommendations provided by the program. Statistical comparisons among groups were performed using the Student’s *t*-test or ANOVA tests as further detailed in each experiment. *p*-values are indicated.

## 3. Results

### 3.1. Flavivirus NS5 Proteins Are Phosphorylated by Human Akt

**Figure 2:**
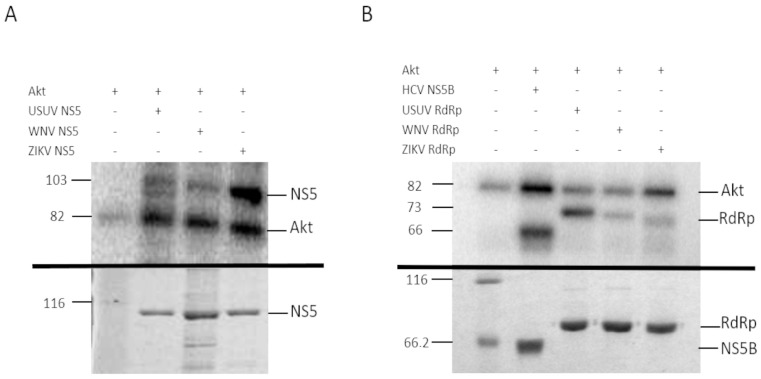
Phosphorylation of USUV, WNV and ZIKV NS5 (**A**) and RdRp domain (**B**) with human recombinant Akt. (**A**) The upper panel shows the in vitro phosphorylation of recombinant USUV, WNV and ZIKV NS5 proteins containing the entire sequence (**A**) or the RdRp domain only (**B**). The phosphorylation reactions were resolved on a 10% SDS-PAGE gel. The band corresponding to the autophosphorylation of Akt is indicated on the right (line Akt, Theoretical MW: 81.2 kDa), and the molecular weights on the left. The bottom panel shows a Coomassie stain of the recombinant proteins used in the assay, after being resolved by SDS-PAGE in a 10% acrylamide gel. Unstained Protein Molecular Weight Marker, Thermo Fisher (lane 1), USUV NS5 (lane 2), WNV NS5 (lane 3) and ZIKV NS5 (lane 4). In vitro phosphorylation of HCV NS5B as positive control is also shown in B. USUV RdRp, WNV RdRp and ZIKV RdRp domain in upper panel. The bottom panel shows the Coomassie stain of the recombinant proteins used in the upper panel, in a 10% SDS-PAGE gel. Unstained Protein Molecular Weight Marker, Thermo Fisher (lane 1), HVC NS5B (lane 2), USUV RdRp (lane 3), WNV RdRp (lane 4) and ZIKV RdRp (lane 5). Expected molecular weights for HCV NS5B, RdRp domains, and NS5s are 66 kDa, 73 kDa, and 103 kDa, respectively. The data shown in each panel is representative of the results obtained from at least three independent experiments.

DNA fragments spanning USUV, WNV, and ZIKV NS5 residues encoding the full-length NS5 protein or its RdRpD were amplified by PCR and cloned into pET-21b/pRSET and pcDNA3 using the forward and reverse primers listed in Table 1 (Figure 1). His-tagged NS5 proteins were overexpressed in *E. coli* and purified by affinity chromatography (Figure 2 bottom panels). The identity of each protein was confirmed by mass spectrometry that at least covered 93% of the entire sequence in all proteins (Supplementary Figure S1).

**Figure 3:**
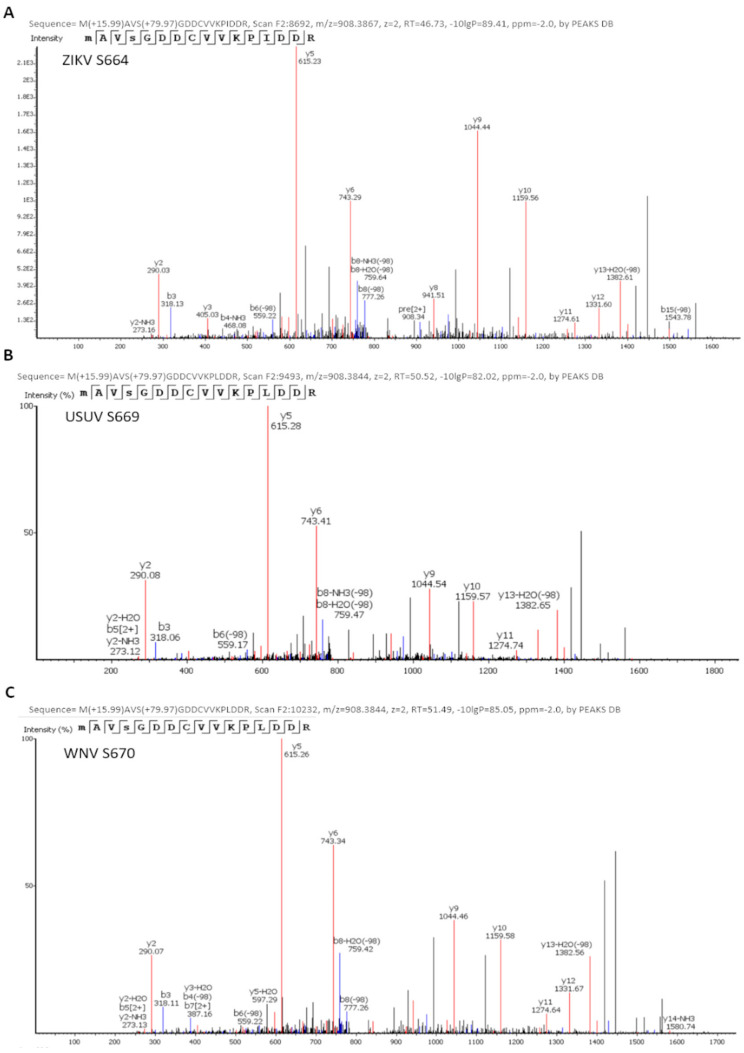
Identification of ZIKV (**A**), USUV (**B**), and WNV (**C**) NS5 phosphorylation sites. Spectra of the phosphopeptides identified by mass spectrometry analyses are shown. The position of the phosphorylated amino acid and the name of the protein analyzed are indicated in the upper left corner of each panel. The theoretical m/z of the phosphorylated peptides is shown, which in this case is 908.38. The theoretical m/z of the unphosphorylated peptides is 868.40. In addition, in each spectrum the series of fragments “y” (in red) and “b” (in blue) are shown, which justify the sequence assignment.

We confirmed that all these recombinant proteins were phosphorylated by Akt in vitro (Figure 2). The phosphorylated positions of band-purified NS5s, including the corresponding to USUV previously described [11], were determined by mass spectrometry, with sequence coverage values of 93%, 95% and 96% for WNV, ZIKV and USUV NS5 proteins, respectively (Supplementary Figure S1). The analysis revealed that the same residue, a Ser contained in the catalytic motif SGDD, was phosphorylated in all flaviviral NS5 proteins tested: S664, S669 and S670 in ZIKV, USUV, and WNV, respectively (Figure 3). In addition, T34 and S167 residues of the MTase domain, and T397 of the RdRpD were also phosphorylated in ZIKV NS5 (Supplementary Figure S2). 

### 3.2. Flavivirus NS5 Proteins Are Co-Immunoprecipitated with Akt

**Figure 4:**
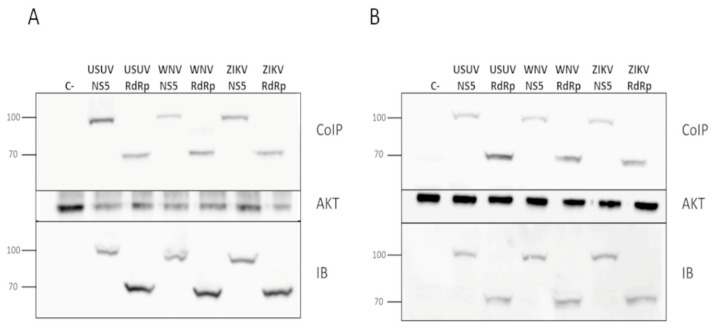
Co-immunoprecipitation of flavivirus NS5 in Akt pull-downs. Plasmids pcDNA3-USUV_NS5, pcDNA3-USUV_RdRp, pcDNA3-WNV_NS5, pcDNA3-ZIKV_NS5, pcDNA3-WNV RdRp or pcDNA3-WNV RdRp were transfected into Huh7.5 (**A**) and Hek-293T cells (**B**). Cell extracts were immunoprecipitated with an anti-Akt antibody, and then immunoblotted using an anti-HA antibody which recognizes the tested viral proteins (upper panel). To confirm the presence of Akt, the same membranes were stripped and blotted again using an anti-Akt antibody, which can be seen on the Akt panel. An aliquot taken from each whole-cell lysate, which were later used in the co-immunoprecipitation experiments, was immunoblotted with anti-HA (IB panel). The molecular weights of marker proteins are indicated on the left. The data in each panel is representative of the results from at least three independent experiments.

We have recently demonstrated that USUV NS5 colocalizes with Akt in a cellular context, and both proteins are pulled down together in Co-IP assays [11]. To elucidate whether ZIKV and WNV NS5 proteins interact with endogenous Akt in a live cell, we performed immunoprecipitation assays as those described above, using two different human cell lines. We confirmed that ZIKV and WNV NS5s also coprecipitate with endogenous Akt, independently of the cell line used (Figure 4). 

### 3.3. RdRp Activity of Wild-Type and Mutant Proteins

**Figure 5:**
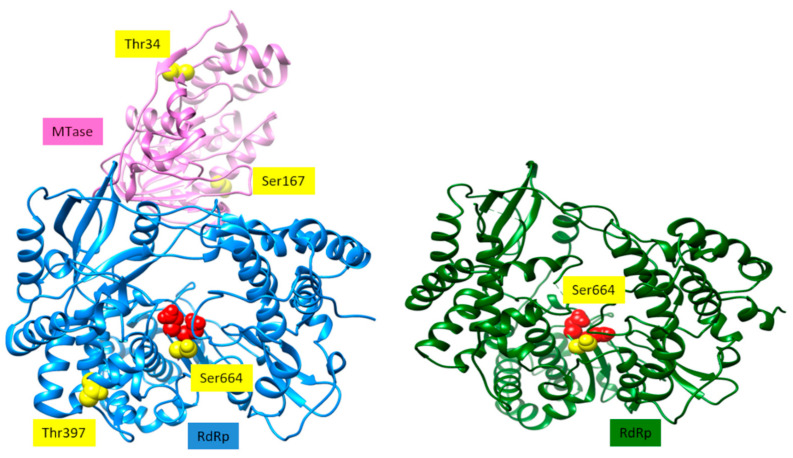
Structure of ZIKV NS5 [22] is shown as ribbons on the left. The MTase domain is shown in pink, and the RdRp domain is in blue. The two aspartates of the catalytic site are shown as red spheres. Sites that can potentially be phosphorylated by Akt/PKB are represented as yellow spheres. Structure of WNV RdRp domain [23] is depicted as ribbons. The two Asp residues of the catalytic site are shown as red spheres. The site that can be potentially phosphorylated by Akt/PKB is depicted as yellow spheres.

**Figure 6:**
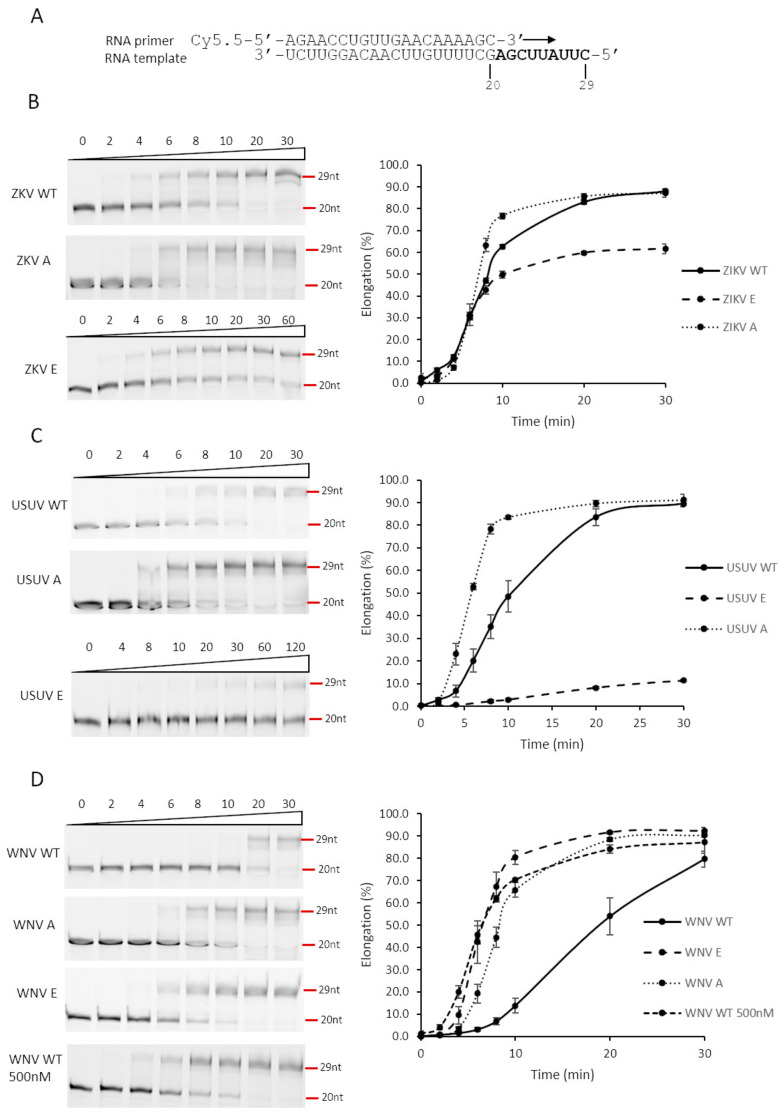
Effect of substitutions of RdRp catalytic site Ser residue on primer extension. (**A**) Primer/Template used in the assay. The primer is a 20-nucleotide RNA molecule which is fluorescently labeled at the 5’ end. The template molecule contains 29 residues. Thus, when primer is hybridized to the template and extended by the polymerase, a fluorescently labeled molecule of 29 nucleotides is expected. (**B**–**D**) ZIKV, USUV, and WNV NS5 proteins were assayed for primer extension, using a fluorescent-based methodology, described in Materials and Methods section. PAGE gels with a representative experiment are shown on the left for each one of the conditions and proteins used. Elongation (primer-extension activity normalized against the total fluorescent signal observed for each line) is represented over time for each one of the proteins assayed (right). Values corresponding to the mean and SEM of at least three independent experiments are represented. ZIKV WT, USUV WT and WNV WT correspond to the original recombinant proteins. ZIKV E, USUV E and WNV E correspond to Ser-to-Glu mutants and ZIKV A, USUV A and WNV A to Ser-to-Ala mutants. The final protein concentration was 100 nM, except in panel D where in one of the experiments it was 500 nM.

Protein phosphorylation is a post-translational modification which is generally involved in modulating the activity of a cellular enzyme, by either switching on or off its activity. Hence, we considered that phosphorylation of viral NS5 in its RdRpD could be affecting its RNA-polymerase activity. Notably, the Ser residue phosphorylated in flaviviral NS5 proteins (Ser664, Ser669 and Ser670 in ZIKV, USUV and WNV, respectively) is in the catalytic center of the RdRpD (a fully conserved SGDD sequence, Supplementary Figure S1 and Figure 5). To analyze the possible impact of phosphorylation, we used Ser-to-Glu mutant proteins (E) in this position, as Glu molecular structure mimics a phosphorylated Ser residue. Ser-to-Ala mutants (A) were also included to analyze the impact of removing Ser side chain in this position. We first measured polymerase activity for each wild-type and mutant polymerase in a primer-extension assay using a fluorescent RNA probe. USUV and ZIKV Ser-to-Glu mutants exhibited abnormal polymerization kinetics when compared to their respective wild-type enzymes (Figure 6A,B). Conversely, Ser-to-Glu WNV NS5 mutant showed an enhanced primer-extension activity. When compared to wild-type USUV and ZIKV NS5 proteins (80% extension at 10 min), wild-type WNV NS5 exhibited delayed primer-extension kinetics (20% extension at 10 min) (Figure 6C). This delay was apparently recovered to normal activity levels (80% extension) in WNV Ser-to-Glu and Ser-to-Ala mutants. Furthermore, WNV NS5 protein primer-extension activity increased at similar levels than the mutated WNV proteins when the protein concentration increased (Figure 6C). 

**Figure 7:**
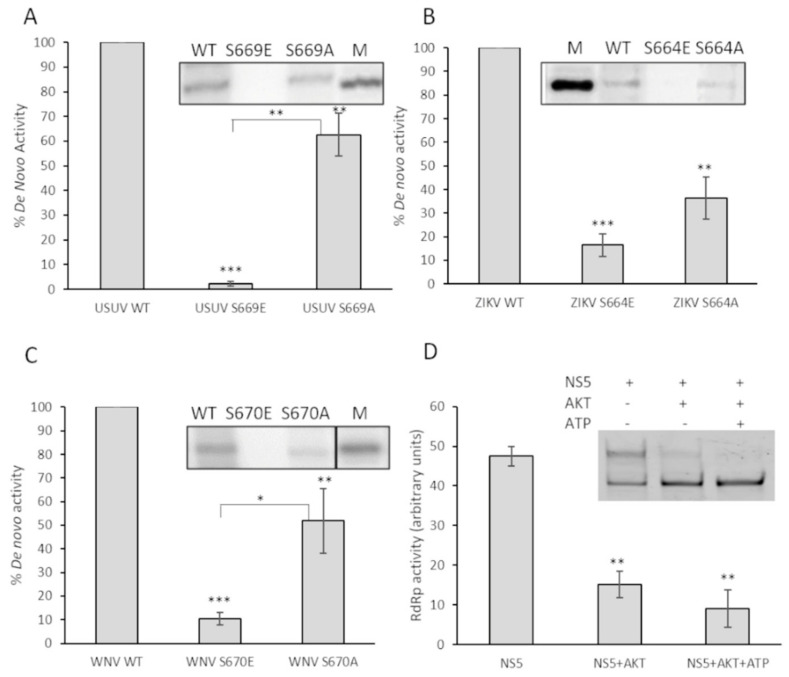
Effect on de novo polymerization activity of substitutions of the Ser located in the catalytic center of the domain. De novo activity of USUV NS5 Ser669 (**A**), ZIKV NS5 Ser664 (**B**), and WNV NS5 Ser670 (**C**) is shown. A total of 200 nM of each enzyme (WT or mutants) were tested as described in Materials and Methods. The graphs represent de novo RdRp activity of the indicated proteins normalized to WT. A representative experiment is shown on the top of each panel. Values shown correspond to the mean and SEM of at least three independent experiments. (**D**) Primer-extension activity of 400 nM wild-type WNV NS5 after 30 min of incubation at room temperature in the presence of active Akt/PKB. After incubation in the presence of Akt, the RNA polymerization reaction was initiated by adding nucleotides and fluorescent T/P. The reaction was allowed to proceed for 6 min at 35 °C. The graph represents relative primer-extension activity with respect to the total fluorescence detected in that sample (100%). A representative experiment is shown on the top of each panel. Values correspond to the mean and SEM of at least three independent experiments. Statistically significant differences (Student’s *t*-test) are represented as follows: * *p* < 0.05; ** *p* < 0.01; *** *p* < 0.001. Asterisks over the bar indicate the *p*-value compared to WT.

To determine if these observations in primer-extension activity correlated with de novo polymerization initiation activity (in the absence of a primer), we followed a different experimental approach using radioisotopes and an oligonucleotide template mimicking the 3’ end of the ZIKV genome. The results clearly show that de novo activity in these mutants was reduced with respect to the wild-type proteins (Figure 7A–C). To ensure that this observation was not due to using too short reaction times, the reaction was allowed to proceed for 90 min. All the Glu mutants showed negligible de novo signal values (Figure 7) even at these time conditions. We next analyzed the effect of the presence of Akt on the primer-extension RNA synthesis reaction by WNV NS5 protein, as described in Figure 6. In the presence of Akt, and irrespectively if ATP is added or not to the assay, a significant decreased in activity is observed (Figure 7D).

### 3.4. Drugs Targeting the PI3K/Akt Route Inhibit Flavivirus Replication in Cell Culture

**Figure 8:**
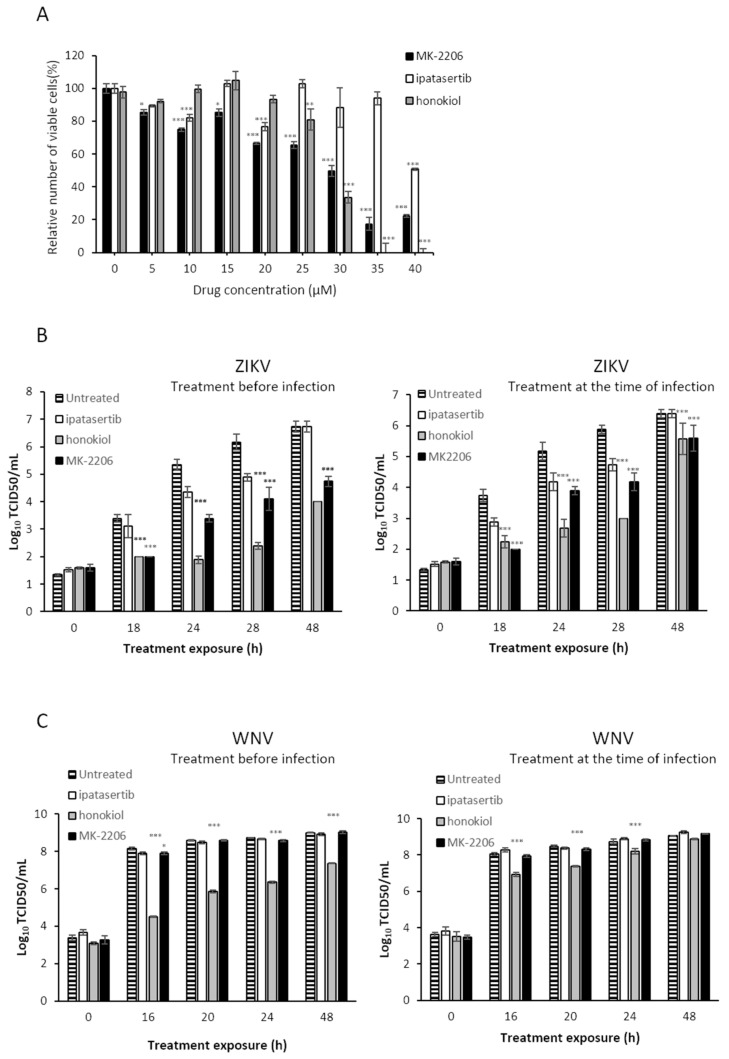
Effect of Akt inhibitors in cell viability (**A**) or in replication kinetics in cell culture (**B**,**C**). (**A**) Percentage of viable cells after treatment with Akt inhibitors for the calculation of CC50. Effect of Akt inhibitors in the replication kinetics of ZIKV (**B**) and WNV (**C**) in cell culture. Virus titer (Log10TCID50/mL or PFU/mL) in the absence or presence of different Akt inhibitors. Inhibitors were added either 5 h before virus infection (left) or at the same time than virus inoculation (right). Viral samples were collected at different time points and titers calculated as described in Materials and Methods. Virus titers recovered from untreated cells, and cells treated with MK-2206, honokiol, and ipatasertib are shown. Values are the average from three independent determinations. Standard deviation values are smaller than the graph symbols for some points and therefore not visible. To determine the statistical significance of the differences observed we have applied two-way ANOVA tests, followed by Dunnett’s correction for multiple comparisons. We have compared each treatment group (MK-2206, honokiol and ipatasertib) to the control group in every time point. Statistically significant differences are represented as follows: * *p* < 0.05; ** *p* < 0.01; *** *p* < 0.001).

To find out if Akt and the PI3K/AKT pathway may be modulating the replication of these flaviviruses we used different specific and non-specific Akt inhibitors. We previously determined the cytotoxicity values for each of the compounds to ensure that the concentration used in the subsequent experiments would ensure at least 80% cell viability (Figure 8A). Thus, the CC50 values calculated for these drugs in Vero cell line were 27 µM, 40 µM, and 28 µM for MK-2206, ipatasertib and honokiol, respectively. Previous results in our laboratory showed that USUV replication kinetics was affected by Akt-specific inhibitors MK-2206 and ipatasertib [11]. We confirmed that these specific drugs also inhibited ZIKV in a similar manner (Figure 8B), but no significant differences were observed for WNV, except for honokiol, a non-specific Akt inhibitor (Figure 8C). We also found that pretreating the cells with these compounds resulted in modestly larger antiviral activities upon ZIKV replication than applying the compounds at the same time than virus inoculations. Of these two Akt-specific drugs, MK-2206 (a non-competitive inhibitor) caused a greater inhibition of viral replication than ipatasertib (a competitive inhibitor). Finally, as we have previously observed with USUV, we found that a well-known non-specific Akt drug that inhibits the PI3K/Akt pathway (honokiol), led to the largest reductions in the viral titers of samples collected from ZIKV- and WNV-infected cells (Figure 8).

## 4. Discussion

Viruses are obligate intracellular parasites that interact with the cellular machinery to ensure their own replication, and to evade the host defenses against infection. To this goal, virally encoded proteins engage in a series of dynamic networks with a vast array of host cellular proteins, altering the normal functioning of the cell. Our laboratory and others have described the role of the cellular kinase Akt and other members of the PI3K/Akt/mTOR pathway, which controls essential cellular functions involved in cell metabolism and proliferation, in the replication of norovirus [24], HCV [10] and USUV [11]. Now we described that the Akt cell kinase phosphorylates the NS5 proteins of ZIKV and WNV. The polymerases of norovirus [24] and HCV (unpublished results) are phosphorylated in residues located in the fingertips domain. Conversely, here we describe that USUV, ZIKV and WNV NS5 proteins are phosphorylated in the Ser residue (SGDD sequence) adjacent to the aspartates which are part of the catalytic triad (Figure 2 and Figure 5). Despite the same residue is phosphorylated in all three NS5 proteins, the effect of phosphorylation on RdRp activity seems to be drastically different. Ser-to-Glu changes (that mimic a phosphorylated Ser) lead to reduced polymerase activities in most viruses analyzed to date (norovirus, HCV, USUV, ZIKV) [10,11,24]. However, the WNV S670E mutant exhibited an enhanced primer-extension activity (e.g., 80% at 10 min), similar to wild-type ZIKV and USUV NS5 proteins analyzed in this work (Figure 6). This apparently contradictory result with respect to other viruses, could be related to a conformational instability of wild-type WNV NS5. In that sense, increments in protein concentration rescued primer extension to levels similar to those observed for wild-type ZIKV and USUV NS5 proteins (Figure 6C). Nevertheless, all Ser-to-Glu NS5 mutants showed nearly undetectable de novo RNA polymerization activity (Figure 7). These results were in accordance with those obtained for primer-extension in the presence of Akt and Akt + ATP for the WNV NS5 protein (Figure 7D), and in agreement with previous results with HCV NS5B [10].

The Ser residue located in NS5 catalytic site (SGDD motif) was the only residue phosphorylated in USUV and WNV. However, we detected other phosphorylated residues in ZIKV NS5. The lack of phosphorylation in the homologous positions in USUV and WNV proteins could be due to absence of a phosphorylatable residue in the homologous position (T34 in ZIKV and I34 in WNV), or lack of an Akt-target sequence in that region (T397, Supplementary Figure S1). Moreover, the analyzed positions may already have a residue that mimics phosphorylation (S167 of ZIKV versus E167 of USUV and WNV). 

The PI3K/Akt pathway controls essential cellular functions involved in cell metabolism and proliferation. Furthermore, Akt has been found to participate in modulating replication in different viruses [25]. The first evidence of this link between PI3K/Akt and viral replication was the discovery in a mouse retrovirus of a viral oncogene encoding for an Akt-like protein which drives cell cycle progression [26]. The way in which viruses interact with PI3K/Akt pathway are diverse. PI3K/Akt can affect virus entry into the cell, viral protein translation and host cell survival, which is a key requirement in persistent infections [27]. The results presented here reveal that Akt may be also playing an important role in modulating flavivirus genome replication. 

The use of drugs against host cell processes to contain virus replication is an alternative antiviral strategy that benefits from a lower likelihood of selecting drug-resistant viruses [6,7]. Here we have investigated the potential antiviral behavior of several specific and non-specific Akt inhibitors on flavivirus infection. We found that viral replication is better inhibited when the cells had been pretreated with the drug. This could be suggesting that early steps in the virus life cycle are affected by the inhibition of Akt. When we compared only those Akt-specific inhibitors, we found that MK-2206, a non-competitive drug [28], works better than ipatasertib, a competitive inhibitor of the ATP binding site at Akt [29]. The conformation of Akt in the presence of different drugs can be affected and may be leading to the stabilization of a closed or an open conformation [30]. Since Akt interacts with NS5 in addition to phosphorylating it, it is conceivable that the interaction itself is relevant to the inhibitory effect observed. Thus, the interaction between NS5 and Akt may be affected in a different manner by Akt-specific inhibitors, leading to the differences observed in viral replication. Future biochemical and structural work is needed to elucidate how these proteins interact with each other, and to fully comprehend the mechanism of action of these drugs upon flavivirus replication.

WNV replication was not affected by any of the specific drugs that inhibit Akt. Only honokiol, a non-specific Akt drug that affects several cellular processes modulated by the PI3K/Akt pathway, elicited a significant inhibition upon virus replication. This could be potentially explained by the lack of effect associated with the Ser-to-Glu change mimicking phosphorylation on RdRp activity (Figure 6). Nonetheless, the complete loss of de novo replication activity in this WNV mutant suggests that there might be other factors involved. Several aspects of WNV interaction with pathways regulating cellular homeostasis may differ from those of USUV and ZIKV. For example, WNV infection is not always strictly dependent on the regulation of autophagy [31,32,33] whereas this pathway is essential for USUV and ZIKV [34,35]. Ongoing work in our lab is aimed at understanding these differences.

Of all Akt drugs tested here, honokiol is the strongest inhibitor for ZIKV replication. This compound has previously shown antiviral activity against DENV [36], HCV [37] and herpes simplex virus type 1 [38]. The mechanism by which honokiol inhibits viral replication remains to be elucidated. Although honokiol is an inhibitor of the PI3K/Akt pathway, there are several other cellular activities affected by this drug, and hence its antiviral behavior could be related to any of them [39]. 

Here we demonstrate that host cell Akt is an interacting partner of flaviviral NS5, and that Akt phosphorylates NS5 because of such interaction. One of the positions phosphorylated is in the catalytic center of the RdRp domain and is fully conserved in all human flaviviruses. NS5 phosphorylation differently affects polymerase activity in different viruses, at least for primer-extension activity. Several Akt drugs inhibit flavivirus replication, and this effect is greater when drugs are administered before infection. These results encourage further studies to investigate the therapeutic value of drugs targeting the PI3K/Akt/mTOR pathway in the treatment of flaviviral disease.

## Figures and Tables

**Table 1:** Primers used in this study.

Name	Sequence (5′->3′) ^1^	5′-End Position ^2^
Zika-RdRp-pRSET-FW	ggcgcatatggttagctgtgccgaagcacc	8465
Zika-pRSET-RV	ggcggaattcctaatgatggtgatgatg	10,370
Zika-NS5-pcDNA-FW	gaattcgccatgtacccatacgatgttccagattacgctggaggtggaacaggagagacc	7667
Zika-pcDNA-RV	ctcgagttacagcactccaggtgtagaccc	10,370
Zika-RdRp-pcDNA-FW	gaattcgccatgtacccatacgatgttccagattacgctgtaagctgcgctgaagctcc	8465
ZIKV-S664E-FW	gaaacgtatggcagtggaaggtgatgattgcgttg	9640
ZIKV-S664E-RV	caacgcaatcatcaccttccactgccatacgtttc	9675
ZIKV-S664A-FW	gaaacgtatggcagtggccggtgatgattgcgttg	9640
ZIKV-S664A-RV	caacgcaatcatcaccggccactgccatacgtttc	9675
WNV-NS5-pET-FW	gctagcatgggtggagccaagggacgcac	7680
WNV-pET-RV	gcggccgcctaatggtgatggtgatggtgcaaaacagtgtcctcaactac	10,394
WNV-RdRp-pET-FW	gctagcatggggaagcctctcctcaattc	8484
WNV-pcDNA-RV	gcggccgcttacaaaacagtgtcctcaactac	10,394
WNV-RdRp-pcDNA-FW	ggtaccgccatgtacccatacgatgttccagattacgctgggaagcctctcctcaattc	8484
WN-NS5-pcDNA-FW	ggtaccgccatgtacccatacgatgttccagattacgctggtggagccaagggacgcac	7680
WNV-S670E-FW	gtcgcatggccgtcgaaggtgatgactgcgtg	9646
WNV-S670E-RV	cacgcagtcatcaccttcgacggccatgcgac	9681
WNV-S670A-FW	gtcgcatggccgtcgccggtgatgactgcgtg	9646
WNV-S670A-RV	cacgcagtcatcaccggcgacggccatgcgac	9681
USUV-S669E-FW	gacccgcatggctgtggaaggagatgattgtgttg	9663
USUV-S669E-RV	caacacaatcatctccttccacagccatgcgggtc	9698
USUV-S669A-RV	caacacaatcatctccagccacagccatgcgggtc	9698
USUV-S669A-FW	gacccgcatggctgtggctggagatgattgtgttg	9663
USUV20	gcucacgcagacgaacgacu	1

^1^ Sequence is in the orientation 5′ to 3′. ^2^ The numbering refers to the strain of ZIKV (GenBank accession number KX377337.1), WNV (GenBank accession number KC407673.1) and USUV (reference sequence NCBI NC_006551.1.).

## Data Availability

The data presented in this study are available on request from the corresponding authors.

## Supplementary Materials

The following are available online at https://www.mdpi.com/article/10.3390/v13050896/s1, Figure S1: NS5 amino acid sequence alignment. Figure S2: Identification of WNV, ZIKV, and USUV NS5 phosphorylation sites.

## References

[1] Pierson T.C., Diamond M.S. (2020). The continued threat of emerging flaviviruses. Nat. Microbiol..

[2] De Clercq E., Li G. (2016). Approved Antiviral Drugs over the Past 50 Years. Clin. Microbiol. Rev..

[3] Eyer L., Nencka R., de Clercq E., Seley-Radtke K., Ruzek D. (2018). Nucleoside analogs as a rich source of antiviral agents active against arthropod-borne flaviviruses. Antivir. Chem. Chemother..

[4] Guarner J., Hale G.L. (2019). Four human diseases with significant public health impact caused by mosquito-borne flaviviruses: West Nile, Zika, dengue and yellow fever. Semin. Diagn. Pathol..

[5] Mas A., Lopez-Galindez C., Cacho I., Gomez J., Martinez M.A. (2010). Unfinished stories on viral quasispecies and Darwinian views of evolution. J. Mol. Biol..

[6] Kumar N., Sharma S., Kumar R., Tripathi B.N., Barua S., Ly H., Rouse B.T. (2020). Host-Directed Antiviral Therapy. Clin. Microbiol. Rev..

[7] Saiz J.C., Oya N.J., Blazquez A.B., Escribano-Romero E., Martin-Acebes M.A. (2018). Host-Directed Antivirals: A Realistic Alternative to Fight Zika Virus. Viruses.

[8] Roesch F., Fajardo A., Moratorio G., Vignuzzi M. (2019). Usutu Virus: An Arbovirus on the Rise. Viruses.

[9] Grant A., Ponia S.S., Tripathi S., Balasubramaniam V., Miorin L., Sourisseau M., Schwarz M.C., Sanchez-Seco M.P., Evans M.J., Best S.M. (2016). Zika Virus Targets Human STAT2 to Inhibit Type I Interferon Signaling. Cell Host Microbe.

[10] Valero M.L., Sabariegos R., Cimas F.J., Perales C., Domingo E., Sanchez-Prieto R., Mas A. (2016). Hepatitis C Virus RNA-Dependent RNA Polymerase Interacts with the Akt/PKB Kinase and Induces Its Subcellular Relocalization. Antimicrob. Agents Chemother..

[11] Albentosa-González L., Sabariegos R., Arias A., Clemente-Casares P., Mas A. (2021). Akt Interacts with Usutu Virus Polymerase, and Its Activity Modulates Viral Replication. Pathogens.

[12] Albentosa-Gonzalez L., Clemente-Casares P., Sabariegos R., Mas A. (2019). Polymerase Activity, Protein-Protein Interaction, and Cellular Localization of the Usutu Virus NS5 Protein. Antimicrob. Agents Chemother..

[13] Lopez-Jimenez A.J., Clemente-Casares P., Sabariegos R., Llanos-Valero M., Bellon-Echeverria I., Encinar J.A., Kaushik-Basu N., Froeyen M., Mas A. (2014). Hepatitis C virus polymerase-polymerase contact interface: Significance for virus replication and antiviral design. Antiviral. Res..

[14] Moreno M.L., Escobar J., Izquierdo-Alvarez A., Gil A., Perez S., Pereda J., Zapico I., Vento M., Sabater L., Marina A. (2014). Disulfide stress: A novel type of oxidative stress in acute pancreatitis. Free Radic. Biol. Med..

[15] Shevchenko A., Wilm M., Vorm O., Mann M. (1996). Mass spectrometric sequencing of proteins silver-stained polyacrylamide gels. Anal. Chem..

[16] Alonso R., Pisa D., Marina A.I., Morato E., Rabano A., Rodal I., Carrasco L. (2015). Evidence for fungal infection in cerebrospinal fluid and brain tissue from patients with amyotrophic lateral sclerosis. Int. J. Biol. Sci..

[17] Jorge I., Casas E.M., Villar M., Ortega-Perez I., Lopez-Ferrer D., Martinez-Ruiz A., Carrera M., Marina A., Martinez P., Serrano H. (2007). High-sensitivity analysis of specific peptides in complex samples by selected MS/MS ion monitoring and linear ion trap mass spectrometry: Application to biological studies. J. Mass Spectrom..

[18] Lu G., Bluemling G.R., Collop P., Hager M., Kuiper D., Gurale B.P., Painter G.R., De La Rosa A., Kolykhalov A.A. (2017). Analysis of Ribonucleotide 5’-Triphosphate Analogs as Potential Inhibitors of Zika Virus RNA-Dependent RNA Polymerase by Using Nonradioactive Polymerase Assays. Antimicrob. Agents Chemother..

[19] Petrovic T., Blazquez A.B., Lupulovic D., Lazic G., Escribano-Romero E., Fabijan D., Kapetanov M., Lazic S., Saiz J. (2013). Monitoring West Nile virus (WNV) infection in wild birds in Serbia during 2012: First isolation and characterisation of WNV strains from Serbia. Eurosurveillance.

[20] Bassi M.R., Sempere R.N., Meyn P., Polacek C., Arias A. (2018). Extinction of Zika Virus and Usutu Virus by Lethal Mutagenesis Reveals Different Patterns of Sensitivity to Three Mutagenic Drugs. Antimicrob. Agents Chemother..

[21] Martin-Acebes M.A., Saiz J.C. (2011). A West Nile virus mutant with increased resistance to acid-induced inactivation. J. Gen. Virol..

[22] Ferrero D.S., Ruiz-Arroyo V.M., Soler N., Uson I., Guarne A., Verdaguer N. (2019). Supramolecular arrangement of the full-length Zika virus NS5. PLoS Pathog..

[23] Malet H., Egloff M.P., Selisko B., Butcher R.E., Wright P.J., Roberts M., Gruez A., Sulzenbacher G., Vonrhein C., Bricogne G. (2007). Crystal structure of the RNA polymerase domain of the West Nile virus non-structural protein 5. J. Biol. Chem..

[24] Eden J.S., Sharpe L.J., White P.A., Brown A.J. (2011). Norovirus RNA-dependent RNA polymerase is phosphorylated by an important survival kinase, Akt. J. Virol..

[25] Dunn E.F., Connor J.H. (2012). HijAkt: The PI3K/Akt pathway in virus replication and pathogenesis. Prog. Mol. Biol. Transl. Sci..

[26] Bellacosa A., Testa J.R., Staal S.P., Tsichlis P.N. (1991). A retroviral oncogene, akt, encoding a serine-threonine kinase containing an SH2-like region. Science.

[27] Diehl N., Schaal H. (2013). Make yourself at home: Viral hijacking of the PI3K/Akt signaling pathway. Viruses.

[28] Hirai H., Sootome H., Nakatsuru Y., Miyama K., Taguchi S., Tsujioka K., Ueno Y., Hatch H., Majumder P.K., Pan B.S. (2010). MK-2206, an allosteric Akt inhibitor, enhances antitumor efficacy by standard chemotherapeutic agents or molecular targeted drugs in vitro and in vivo. Mol. Cancer Ther..

[29] Blake J.F., Xu R., Bencsik J.R., Xiao D., Kallan N.C., Schlachter S., Mitchell I.S., Spencer K.L., Banka A.L., Wallace E.M. (2012). Discovery and preclinical pharmacology of a selective ATP-competitive Akt inhibitor (GDC-0068) for the treatment of human tumors. J. Med. Chem..

[30] Cherrin C., Haskell K., Howell B., Jones R., Leander K., Robinson R., Watkins A., Bilodeau M., Hoffman J., Sanderson P. (2010). An allosteric Akt inhibitor effectively blocks Akt signaling and tumor growth with only transient effects on glucose and insulin levels in vivo. Cancer Biol. Ther..

[31] Beatman E., Oyer R., Shives K.D., Hedman K., Brault A.C., Tyler K.L., Beckham J.D. (2012). West Nile virus growth is independent of autophagy activation. Virology.

[32] Blazquez A.B., Martin-Acebes M.A., Saiz J.C. (2014). Amino acid substitutions in the non-structural proteins 4A or 4B modulate the induction of autophagy in West Nile virus infected cells independently of the activation of the unfolded protein response. Front. Microbiol..

[33] Vandergaast R., Fredericksen B.L. (2012). West Nile virus (WNV) replication is independent of autophagy in mammalian cells. PLoS ONE.

[34] Blazquez A.B., Escribano-Romero E., Merino-Ramos T., Saiz J.C., Martin-Acebes M.A. (2013). Infection with Usutu virus induces an autophagic response in mammalian cells. PLoS Negl. Trop. Dis..

[35] Hamel R., Dejarnac O., Wichit S., Ekchariyawat P., Neyret A., Luplertlop N., Perera-Lecoin M., Surasombatpattana P., Talignani L., Thomas F. (2015). Biology of Zika Virus Infection in Human Skin Cells. J. Virol..

[36] Fang C.Y., Chen S.J., Wu H.N., Ping Y.H., Lin C.Y., Shiuan D., Chen C.L., Lee Y.R., Huang K.J. (2015). Honokiol, a Lignan Biphenol Derived from the Magnolia Tree, Inhibits Dengue Virus Type 2 Infection. Viruses.

[37] Lan K.H., Wang Y.W., Lee W.P., Lan K.L., Tseng S.H., Hung L.R., Yen S.H., Lin H.C., Lee S.D. (2012). Multiple effects of Honokiol on the life cycle of hepatitis C virus. Liver Int..

[38] Liu S., Li L., Tan L., Liang X. (2019). Inhibition of Herpes Simplex Virus-1 Replication by Natural Compound Honokiol. Virol. Sin..

[39] Amblard F., Govindarajan B., Lefkove B., Rapp K.L., Detorio M., Arbiser J.L., Schinazi R.F. (2007). Synthesis, cytotoxicity, and antiviral activities of new neolignans related to honokiol and magnolol. Bioorg. Med. Chem. Lett..

