# Supplementary material for: Akt Kinase Intervenes in Flavivirus Replication by Interacting with Viral Protein NS5
Source: Viruses. 2021 May 12;13(5):896. doi: 10.3390/v13050896 (PMC8151281; doi:10.3390/v13050896)
Supplement: Supplementary file 1 [file viruses-13-00896-s001.zip › viruses-1173758-supplementary.pdf]

```
WNV     GRTLGEVWKERLNQMTKEEFTRYRKEAIIEVDRSAAKHARKEGNVTGGHPVSRGTAKLRW
USUV    GRTLGEQWKEKLNGLSKEDFLKYRKEAITEVDRSAARKARRDGNKTGGHPVSRGSAKLRW
ZIKA    GETLGEKWKARLNQMSALEFYSYKKSGITEVCREEARRALKDGVATGGHAVSRGSAKLRW
        *.**** ** :** ::  :*  *:*..* ** *. *::* ::*  ****.****:*****
WNV     LVERRFLEPVGKVIDLGCGRGGWCYYMATQKRVQEVRGYTKGGPGHEEPQLVQSYGWNIV
USUV    MVERQFVKPIGKVVDLGCGRGGWSYYAATLKGVQEVRGYTKGGPGHEEPMLMQSYGWNLV
ZIKA    LVERGYLQPYGKVIDLGCGRGGWSYYAATIRKVQEVKGYTKGGPGHEEPMLVQSYGWNIV
        :*** :::* ***:*********.** ** : ****:************ *:******:*
WNV     TMKSGVDVFYRPSECCDTLLCDIGESSSSAEVEEHRTVRVLEMVEDWLHRGPKEFCIKVL
USUV    TMKSGVDVYYKPSEPCDTLFCDIGESSSSAEVEEQRTLRILEMVSDWLQRGPREFCIKVL
ZIKA    RLKSGVDVFHMAAEPCDTLLCDIGESSSSPEVEEARTLRVLSMVGDWLEKRPGAFCIKVL
         :******:: .:* ****:*********.**** **:*:*.** ***.: *  **:***
WNV     CPYMPKVIEKMELLQRRYGGGLVRNPLSRNSTHEMYWVSRASGNVVHSVNMTSQVLLGRM
USUV    CPYMPRVMERLEVLQRRYGGGLVRVPLSRNSNHEMYWVSGAAGNIVHAVNMTSQVLIGRM
ZIKA    CPYTSTMMETLERLQRRYGGGLVRVPLSRNSTHEMYWVSGAKSNTIKSVSTTSQLLLGRM
        *** . ::* :* *********** ******.******* * .* :::*. ***:*:***
                                    MTase--RdRPD
WNV     EKRTWKGPQYEEDVNLGSGTRAVGKPLLNSDTSKIKNRIERLKKEYSSTWHHDENHPYRT
USUV    EKRTWHGPKYEEDVNLGSGTRAVGKPQPHTNQEKIKARIQRLKEEYAATWHHDKDHPYRT
ZIKA    DG-PRRPVKYEEDVNLGSGTRAVVSCAEAPNMKIIGNRIERIRSEHAETWFFDENHPYRT
        :  . :  :************** .    .: . *  **:*:: *:: **..*::*****
WNV     WNYHGSYDVKPTGSASSLVNGVVRLLSKPWDTITNVTTMAMTDTTPFGQQRVFKEKVDTK
USUV    WTYHGSYEVKPTGSASSLVNGVVRLMSKPWDAILNVTTMAMTDTTPFGQQRVFKEKVDTK
ZIKA    WAYHGSYEAPTQGSASSLINGVVRLLSKPWDVVTGVTGIAMTDTTPYGQQRVFKEKVDTR
        * *****:. . ******:******:*****.: .** :*******:************:
WNV     APEPPEGVKYVLNETTNWLWAFLARDKKPRMCSREEFIRKVNSNAALGAMFEEQNQWRSA
USUV    APEPPSGVREVMDETTNWLWAFLAREKKPRLCTREEFKRKVNSNAALGAMFEEQNQWSSA
ZIKA    VPDPQEGTRQVMSMVSSWLWKELGKHKRPRVCTKEEFINKVRSNAALGAIFEEEKEWKTA
        .*:* .*.: *:. .:.***  *.:.*:**:*::*** .**.*******:***:::* :*
WNV     REAVEDPKFWEMVDEEREAHLRGECHTCIYNMMGKREKKPGEFGKAKGSRAIWFMWLGAR
USUV    REAVEDPRFWEMVDEERENHLKGECHTCIYNMMGKREKKLGEFGKAKGSRAIWFMWLGAR
ZIKA    VEAVNDPRFWALVDKEREHHLRGECQSCVYNMMGKREKKQGEFGKAKGSRAIWYMWLGAR
         ***:**:** :**:*** **:***::*:********** *************:******
WNV     FLEFEALGFLNEDHWLGRKNSGGGVEGLGLQKLGYILREVGTRPGGKIYADDTAGWDTRI
USUV    FLEFEALGFLNEDHWLGRKNSGGGVEGLGVQKLGYILREMSHHSGGKMYADDTAGWDTRI
ZIKA    FLEFEALGFLNEDHWMGRENSRGGVEGLGLQRLGYVLEEMSRIPGGRMYADDTAGWDTRI
        ***************:**:** *******:*:***:*.*:.  .**::************
WNV     TRADLENEAKVLELLDGEHRRLARAIIELTYRHKVVKVMRPAADGRTVMDVISREDQRGS
USUV    TRADLDNEAKVLELMEGEHRQLARAIIELTYKHKVVKVMRPGTDGKTVMDVISREDQRGS
ZIKA    SRFDLENEALITNQMEKGHRALALAIIKYTYQNKVVKVLRPAEKGKTVMDIISRQDQRGS
        :* **:*** : : ::  ** ** ***: **::*****:**. .*:****:***:*****
WNV     GQVVTYALNTFTNLAVQLVRMMEGEGVIGPDDVEKLTKGKGPKVRTWLFENGEERLSRMA
USUV    GQVVTYALNTFTNIAVQLIRLMEAEGVIGQEHLESLPRKTKYAVRTWLFENGEERVTRMA
ZIKA    GQVVTYALNTFTNLVVQLIRNMEAEEVLEMQDLWLLRRSE--KVTNWLQSNGWDRLKRMA
        *************:.***:* **.* *:  :.:  * :     * .** .** :*:.***
WNV     VSGDDCVVKPLDDRFATSLHFLNAMSKVRKDIQEWKPSTGWYDWQQVPFCSNHFTELIMK
USUV    VSGDDCVVKPLDDRFANALHFLNSMSKVRKDVPEWKPSSGWHDWQQVPFCSNHFQELIMK
ZIKA    VSGDDCVVKPIDDRFAHALRFLNDMGKVRKDTQEWKPSTGWDNWEEVPFCSHHFNKLHLK
        **********:***** :*:*** *.*****  *****:** :*::*****:** :* :*
WNV     DGRTLVVPCRGQDELVGRARISPGAGWNVRDTACLAKSYAQMWLLLYFHRRDLRLMANAI
USUV    DGRTLVVPCRGQDELIGRARVSPGSGWNVRDTACLAKAYAQMWLLLYFHRRDLRLMANAI
ZIKA    DGRSIVVPCRHQDELIGRARVSPGAGWSIRETACLAKSYAQMWQLLYFHRRDLRLMANAI
        ***::***** ****:****:***:**.:*:******:***** ****************
WNV     CSAVPVNWVPTGRTTWSIHAGGEWMTTEDMLEVWNRVWIEENEWMEDKTPVEKWSDVPYS
USUV    CSAVPSNWVPTGRTSWSVHATGEWMTTDDMLEVWNKVWIQDNEWMLDKTPVQSWTDIPYT
ZIKA    CSSVPVDWVPTGRTTWSIHGKGEWMTTEDMLVVWNRVWIEENDHMEDKTPVTKWTDIPYL
        **:** :*******:**:*. ******:*** ***:***::*: * ***** .*:*:**
WNV     GKREDIWCGSLIGTRTRATWAENIQVAINQVRAIIGDE-KYVDYMSSLKRYEDTTLVEDT
USUV    GKREDIWCGSLIGTRTRATWAENIYAAINQVRAIIGQE-KYRDYMLSLRRYEEVNVQEDR
ZIKA    GKREDLWCGSLIGHRPRTTWAENIKNTVNMMRRIIGDEEKYMDYLSTQVRYLGEEGSTPG
        *****:******* *.*:******  ::* :* ***:* ** **: :  **
WNV     VL
USUV    VL
ZIKA    VL
        **
```

Supplementary Figure S1. NS5 amino acid sequence alignment. Amino acid alignment of the NS5 protein sequences of WNV, USUV and ZIKV. Yellow shading shows the sequence that was covered by the proteomics analysis for each protein. Amino acids that are phosphorylated by Akt are shown in red. Asterisks indicate fully conserved positions and colons indicate a high degree of conservation in the detected changes. The sequence motif separating the MTase and RdRP domains is indicated.

A

Sequence= SGIT(+79.97)EVCREEAR, Scan F2:6577, m/z=715.3090, z=2, RT=36.14, -10lgP=63.30, ppm=-2.4, by PEAKS DB

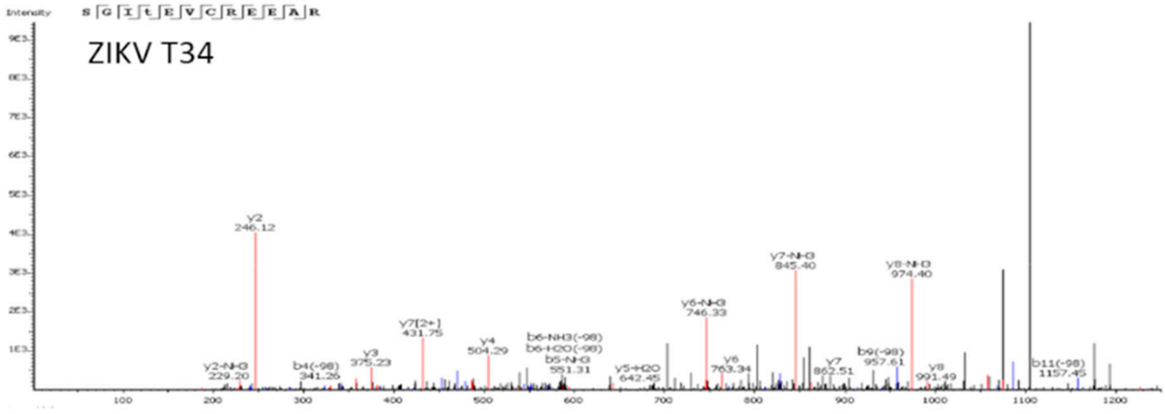

B

Sequence= VLS(+79.97)M(+15.99)VGDWLEK, Scan F2:16403, m/z=686.8188, z=2, RT=89.78, -10lgP=65.87, ppm=6.3, by PEAKS DB

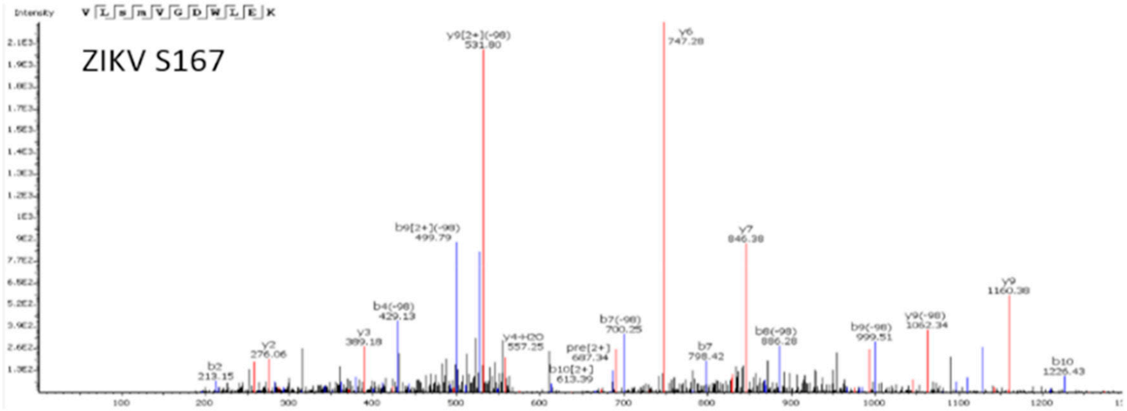

C

Sequence= VCT(+79.97)KEEFINK, Scan F2:7032, m/z=645.7916, z=2, RT=38.39, -10lgP=64.00, ppm=-3.0, by PEAKS DB

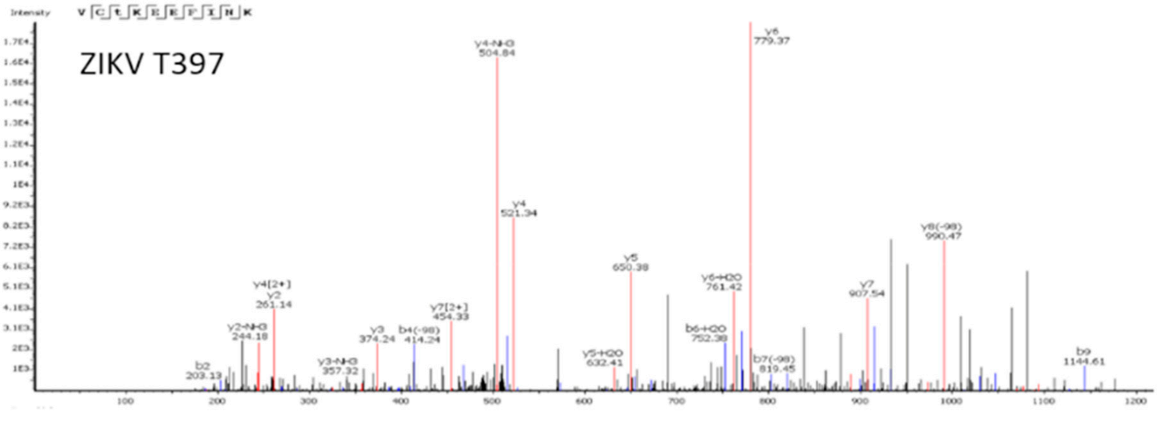

Supplementary Figure S2. Identification of ZIKV NS5 phosphorylation sites. Spectra of the phosphopeptides identified by mass spectrometry analyses are shown. The position of the phosphorylated amino acid and the name of the protein analyzed are indicated in the upper left corner of each panel. The theoretical m/z of the phosphorylated peptides is shown, which in this case is 715.31, 686.82, and 645.79 for T34, S167, and T397, respectively. The theoretical m/z of the unphosphorylated peptides are 675.32, 646.83, and 605.81, respectively. In addition, in each spectrum the series of fragments "y" (in red) and "b" (in blue) are shown, which justify the sequence assignment.
